# Supplementary material for: Multi‐dataset identification of innovative feature genes and molecular mechanisms in keratoconus
Source: J Cell Mol Med. 2024 Sep 19;28(18):e70079. doi: 10.1111/jcmm.70079 (PMC11412914; doi:10.1111/jcmm.70079)
Supplement: Supplementary file 2 — Material S2. [file JCMM-28-e70079-s002.zip › 7.GSEA analysis/5.RNF217-AS1/KEGG_DRUG_METABOLISM_OTHER_ENZYMES.html]

Details for gene set KEGG\_DRUG\_METABOLISM\_OTHER\_ENZYMES[GSEA]

|  || Dataset | RNF217-AS1.group.cls#h\_versus\_l |
| Phenotype | group.cls#h\_versus\_l |
| Upregulated in class | h |
| GeneSet | KEGG\_DRUG\_METABOLISM\_OTHER\_ENZYMES |
| Enrichment Score (ES) | 0.22719452 |
| Normalized Enrichment Score (NES) | 0.70475876 |
| Nominal p-value | 0.93690246 |
| FDR q-value | 0.9616513 |
| FWER p-Value | 1.0 |
Table: GSEA Results Summary

  

Fig 1: Enrichment plot: KEGG\_DRUG\_METABOLISM\_OTHER\_ENZYMES      
 Profile of the Running ES Score & Positions of GeneSet Members on the Rank Ordered List

  

| SYMBOL | TITLE | RANK IN GENE LIST | RANK METRIC SCORE | RUNNING ES | CORE ENRICHMENT || 1 | UGT1A8 | na | 901 | 0.455 | 0.0559 | Yes |
| 2 | UPP2 | na | 1406 | 0.401 | 0.1136 | Yes |
| 3 | UGT2B4 | na | 3115 | 0.286 | 0.1159 | Yes |
| 4 | CYP3A43 | na | 3651 | 0.265 | 0.1483 | Yes |
| 5 | UGT1A6 | na | 4124 | 0.248 | 0.1794 | Yes |
| 6 | UPB1 | na | 4405 | 0.237 | 0.2141 | Yes |
| 7 | UGT2B11 | na | 5963 | 0.184 | 0.2024 | Yes |
| 8 | UPP1 | na | 6611 | 0.164 | 0.2134 | Yes |
| 9 | UGT2B28 | na | 7074 | 0.151 | 0.2272 | Yes |
| 10 | CYP3A4 | na | 9231 | 0.100 | 0.1831 | No |
| 11 | DPYS | na | 9307 | 0.099 | 0.1988 | No |
| 12 | UGT2B10 | na | 9434 | 0.097 | 0.2126 | No |
| 13 | UGT2A3 | na | 10034 | 0.086 | 0.2108 | No |
| 14 | UGT2A1 | na | 14187 | 0.028 | 0.0962 | No |
| 15 | TYMP | na | 14474 | 0.025 | 0.0925 | No |
| 16 | CES2 | na | 14922 | 0.020 | 0.0832 | No |
| 17 | CYP2A7 | na | 15062 | 0.018 | 0.0825 | No |
| 18 | IMPDH1 | na | 15524 | 0.014 | 0.0717 | No |
| 19 | ITPA | na | 16864 | -0.000 | 0.0331 | No |
| 20 | UMPS | na | 19067 | -0.021 | -0.0267 | No |
| 21 | GMPS | na | 19523 | -0.025 | -0.0352 | No |
| 22 | IMPDH2 | na | 19623 | -0.026 | -0.0333 | No |
| 23 | XDH | na | 19625 | -0.026 | -0.0286 | No |
| 24 | GUSB | na | 19854 | -0.029 | -0.0300 | No |
| 25 | TPMT | na | 19905 | -0.029 | -0.0262 | No |
| 26 | UCK1 | na | 20107 | -0.031 | -0.0265 | No |
| 27 | UCKL1 | na | 20280 | -0.033 | -0.0255 | No |
| 28 | NAT1 | na | 20528 | -0.035 | -0.0263 | No |
| 29 | CDA | na | 22365 | -0.055 | -0.0694 | No |
| 30 | CYP2A13 | na | 22404 | -0.055 | -0.0606 | No |
| 31 | TK1 | na | 23790 | -0.072 | -0.0875 | No |
| 32 | CYP3A7 | na | 24059 | -0.076 | -0.0815 | No |
| 33 | HPRT1 | na | 24295 | -0.080 | -0.0739 | No |
| 34 | UCK2 | na | 24949 | -0.089 | -0.0766 | No |
| 35 | UGT2B7 | na | 26756 | -0.121 | -0.1069 | No |
| 36 | UGT2B15 | na | 26916 | -0.124 | -0.0892 | No |
| 37 | CES5A | na | 27987 | -0.148 | -0.0933 | No |
| 38 | TK2 | na | 29905 | -0.203 | -0.1119 | No |
| 39 | CES1 | na | 31598 | -0.269 | -0.1123 | No |
| 40 | CYP3A5 | na | 33128 | -0.370 | -0.0897 | No |
| 41 | NAT2 | na | 33159 | -0.373 | -0.0234 | No |
| 42 | DPYD | na | 33263 | -0.381 | 0.0422 | No |
Table: GSEA details [plain text format]

  

Fig 2: KEGG\_DRUG\_METABOLISM\_OTHER\_ENZYMES      
 Blue-Pink O' Gram in the Space of the Analyzed GeneSet

  

Fig 3: KEGG\_DRUG\_METABOLISM\_OTHER\_ENZYMES: Random ES distribution      
 Gene set null distribution of ES for **KEGG\_DRUG\_METABOLISM\_OTHER\_ENZYMES**
